# DNA methylation in *PRDM8* is indicative for dyskeratosis congenita

**DOI:** 10.18632/oncotarget.7458

**Published:** 2016-02-17

**Authors:** Carola I. Weidner, Qiong Lin, Carina Birkhofer, Uwe Gerstenmaier, Andrea Kaifie, Martin Kirschner, Heiko Bruns, Stefan Balabanov, Arne Trummer, Clemens Stockklausner, Britta Höchsmann, Hubert Schrezenmeier, Marcin Wlodarski, Jens Panse, Tim H. Brümmendorf, Fabian Beier, Wolfgang Wagner

**Affiliations:** ^1^ Helmholtz-Institute for Biomedical Engineering, RWTH Aachen University Medical Faculty, Aachen, Germany; ^2^ Institute for Biomedical Technology – Cell Biology, RWTH University Medical School, Aachen, Germany; ^3^ Varionostic GmbH, Ulm, Germany; ^4^ Department of Hematology, Oncology, Hemostaseology and Stem Cell Transplantation, RWTH Aachen University Medical Faculty, Aachen, Germany; ^5^ Department of Internal Medicine 5-Hematology/Oncology, University Hospital Erlangen, Erlangen, Germany; ^6^ Division of Hematology, University Hospital Zurich, Zurich, Switzerland; ^7^ Department of Hematology, Hemostasis, Oncology, and Stem Cell Transplantation, Hannover Medical School, Hannover, Germany; ^8^ Department of Pediatric Oncology, Hematology and Immunology, University of Heidelberg, Heidelberg, Germany; ^9^ Institute of Transfusion Medicine, University of Ulm, Ulm, Germany; ^10^ Institute of Clinical Transfusion Medicine and Immunogenetics, German Red Cross Blood Transfusion Service Baden-Württemberg-Hessen and University Hospital Ulm, Ulm, Germany; ^11^ Department of Pediatrics, Hematology and Oncology, University of Freiburg, Freiburg, Germany

**Keywords:** aplastic anemia, bone marrow failure, DNA methylation, dyskeratosis congenita, epigenetic, Gerotarget

## Abstract

Dyskeratosis congenita (DKC) is associated with impaired telomere maintenance and with clinical features of premature aging. In this study, we analysed global DNA methylation (DNAm) profiles of DKC patients. Age-associated DNAm changes were not generally accelerated in DKC, but there were significant differences to DNAm patterns of healthy controls, particularly in CpG sites related to an internal promoter region of PR domain containing 8 (*PRDM8*). Notably, the same genomic region was also hypermethylated in aplastic anemia (AA) – another bone marrow failure syndrome. Site-specific analysis of DNAm level in *PRDM8* with pyrosequencing and MassARRAY validated aberrant hypermethylation in 11 DKC patients and 27 AA patients. Telomere length, measured by flow-FISH, did not directly correlate with DNAm in *PRDM8*. Therefore the two methods may be complementary to also identify patients with still normal telomere length. In conclusion, blood of DKC patients reveals aberrant DNAm patterns, albeit age-associated DNAm patterns are not generally accelerated. Aberrant hypermethylation is particularly observed in *PRDM8* and this may support identification and classification of bone marrow failure syndromes.

## INTRODUCTION

Dyskeratosis congenita (DKC) is a rare disease that is often associated with mutations in genes required for proper telomere maintenance (e.g*. DKC1, TERT, RTEL1, TIN2, TERC*) [1, 2]. It was the first disease linked to short telomeres [3]. Clinically, DKC usually reflects a triad of oral leukoplakia, nail dystrophy, and skin hyperpigmentation. Other typical manifestations include symptoms of bone marrow failure, and fibrosis of lung and liver [4]. Diagnosis of DKC necessitates awareness in the clinic, but also appropriate laboratory testing of telomere length (TL; usually below the 1% percentile) and genetic sequencing [5, 6]. However, not all DKC patients reveal dramatically shortened telomeres [7] and clinically relevant mutations are not always to be found [8]. Acquired aplastic anemia (AA) is another bone marrow failure syndrome that is likewise characterized by telomere attrition [4, 9]. Correct diagnosis of DKC and AA is important for adequate treatment and more specific biomarkers are urgently wanted [4]. The advances in epigenetic profiling methods provide new perspectives for biomarkers development [10]. Specific DNA methylation (DNAm) changes are indicative for diagnosis, prognosis, choice of therapeutic regimen in many diseases [11]. In this study, we analyzed DNAm profiles of DKC patients to further clarify if age-associated DNAm changes are enhanced in this disease [12] and if aberrant DNAm patterns can be used to support diagnosis of DKC.

## RESULTS AND DISCUSSION

**Figure 1:**
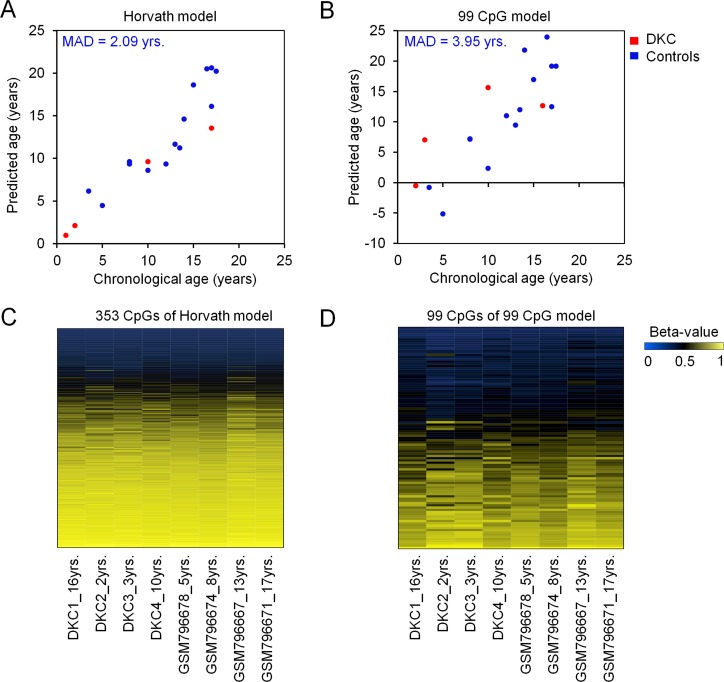
Epigenetic age-predictions of DKC samples Biological age was estimated in DNAm profiles of four DKC samples (red; whole blood) and healthy control samples (blue; here 14 samples are exemplarily depicted from GSE32148) [33] using a model for epigenetic age-predictions by Horvath **A.** [13], or our previously published model based on 99 age-associated CpGs **B.** [12, 14] [46]. The mean absolute deviation of predicted and chronological age (MAD) is indicated for the control samples. Heatmaps of the 353 age-associated CpGs of the Horvath model **C.** and the 99 age-related CpGs of our age-prediction model **D.** demonstrate similar DNAm patterns of DKCs patients and controls. Although DKC is considered to be a premature aging syndrome these broader signatures did not reveal the epigenetic age-acceleration in DKC that we previously described for three age-associated CpGs [12].

Global DNAm profiles of peripheral blood of four DKC patients were analyzed by Illumina HumanMethylation450 BeadChip technology. We utilized two models for epigenetic age-predictions: a model developed by Horvath that is based on DNAm at 353 age-associated CpG dinucleotides [13], and our previously published model based on 99 age-associated CpGs [12, 14][]. Overall, the epigenetic age-predictions did not demonstrate general acceleration of age-associated DNAm (Figure 1). This was unexpected, as we previously observed overestimation of biological age using an epigenetic signature based on only three age-associated CpG sites [12]. This discrepancy may partly be attributed to the fact that the linear model for the three-CpG-Signature has not been specifically trained for young donors and to the possibility that only specific CpGs reveal aberrant DNAm in DKC. Genes that are frequently mutated in DKC (such as *DKC1*, *TINF2, TERT*, *TERC*, and *RTEL1*) did not reflect aberrant DNAm patterns (Supplemental Figure 1).

**Figure 2:**
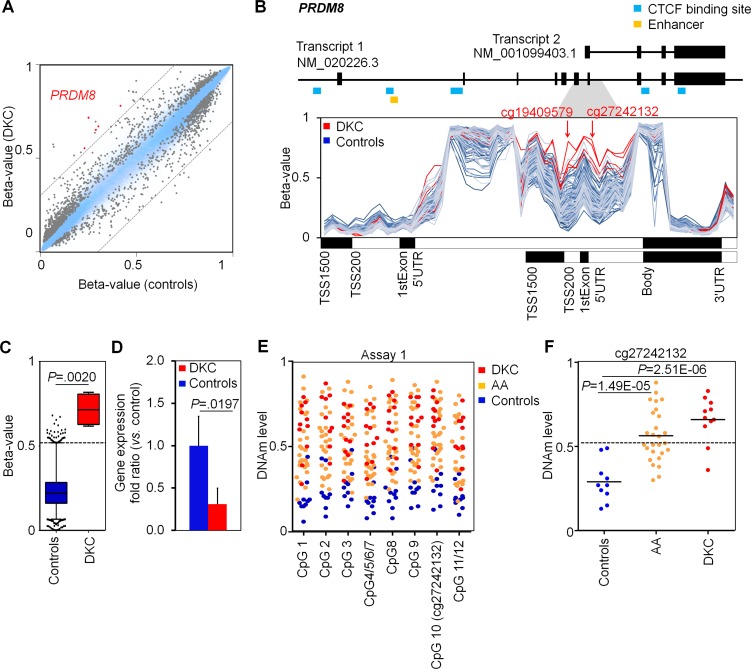
Dyskeratosis congenita is associated with hypermethylation in *PRDM8* **A.** Scatter plots of mean DNAm levels (four DKC patients as compared to four age and gender matched controls) depict 764 hypo- and 1,369 hyper-methylated CpGs in DKC (adjusted *P* value < 0.05; relevant CpGs of *PRDM8* are indicated in red). **B.** DNAm levels (beta-values) of CpGs associated with *PRDM8* reflect hypermethylation at an internal promoter region in DKC patients (as compared to DNAm profiles of normal blood). The positions of two relevant CpGs are indicated (cg19409579 and cg27242132). **C.** Boxplots represent distributions of beta-values at cg27242132 in four DKC samples and 4,131 DNAm profiles of blood samples of 16 different studies (see also supplemental Figure 3). The 99 percentile of controls is indicated as dotted line (DNAm level of 52%). **D.** Quantitative RT-PCR reveals moderate down-regulation of *PRDM8* expression in DKC patients (*n* = 5; two-sided Student's *t*-test; *P* = 0.0197). **E.** MassARRAY analysis of DNAm at cg27242132 (*PRDM8*) revealed consistent DNAm levels in neighboring CpGs (controls: *n* = 10; AA: *n* = 27; DKC: *n* = 11). **F.** In comparison to normal controls the DNAm levels at the CpG site cg27242132 were significantly higher in AA (*P* = 1.49E-05) and particularly in DKC patients (*P* = 2.51E-06).

Comparison of DNAm profiles with four age and gender matched controls revealed 764 hypo- and 1,369 hypermethylated CpGs in DKC (adjusted *P* value < 0.05; Supplemental Table 1). Hypermethylated CpGs were highly significantly enriched in promoter regions and CpG islands (Supplemental Figure 2). These results led us to the assumption, that specific DNAm patterns can be used to support diagnosis of DKC. Therefore, we focused on CpGs with a difference in mean DNAm levels of at least 30%: 26 CpGs reached this cutoff including five in the gene PR domain containing 8 (*PRDM8*) and two in *PRDM16* (Figure 2A, Supplemental Table 2). It has been demonstrated that PRDM8 is implicated in axon outgrowth [15, 16] and regulation of testis steroidogenesis in mice [17] - but hardly anything is known about its potential role in blood formation. In contrast, PRDM16 is involved in human leukemic translocations and was shown to be a physiologic regulator of hematopoietic stem cells [18-20]. Notably, PRDM8 and PRDM16 comprise zinc finger motifs as well as PR domains with methyltransferase activity (H3K9) [16, 17] making them potential epigenetic modifiers of the histone code.

Hypermethylation in PRDM8 was particularly observed at the CpG sites cg27242132 and cg19409579 corresponding to an internal gene region that was hardly methylated in DNAm profiles of normal blood (analyzed in 4,131 DNAm profiles; Figure 2B and 2C). DNAm levels at this region were rather independent from donor age (R^2^ = 0.0311; Supplemental Figure 3), whereas the cellular composition may be relevant as *PRDM8* is slightly higher methylated in lymphocytes than in granulocytes or monocytes (Supplemental Figure 4) [21, 22]. The aberrantly higher DNAm in *PRDM8* was also reflected on lower gene expression level of *PRDM8* in qRT-PCR analysis (Figure 2D).

For site-specific analysis of DNAm at *PRDM8* we designed pyrosequencing assays for the CpGs cg27242132 (assay 1) and cg19409579 (assay 2). However, the measurements were susceptible for the annealing temperature in PCR (Supplemental Figure 5) and therefore we developed alternative assays based on MassARRAY (Figure 2E and 2F, Supplemental Figure 6). MassARRAY results were very similar to HumanMethylation450 BeadChip data. Analysis of 11 DKC samples demonstrated significantly higher DNAm levels as compared to normal controls (*P* = 2.51E-06; Figure 2E and 2F). Notably, blood samples of 27 AA patients were also hypermethylated at this region (*P* = 1.49E-05), suggesting that DNAm in *PRDM8* could serve as a new indicator for bone marrow failure syndromes. As cutoff for normal DNAm level at the CpG site cg27242132 we adopted the 99% percentile of the 4,131 DNAm profiles of normal blood, corresponding to a DNAm level of 52% (Figure 2D): Using this cutoff 81.8% of the DKC (9 of 11) and 51.9% AA patients (14 of 27) were identified as positive.

**Figure 3:**
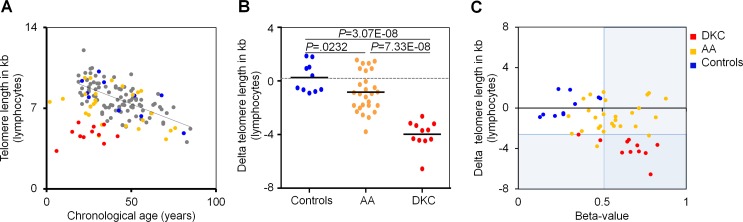
DNAm in *PRDM8* is indicative for DKC and AA **A.** Telomere length of lymphocytes was measured by flow-FISH for 114 healthy individuals (grey and blue) [12], 11 dyskeratosis congenital patients (DKC; red) and 27 patients with aplastic anemia (AA; yellow). **B.** Delta telomere length - in relation to age-adjusted mean telomere length in 104 healthy controls - revealed telomere attrition particularly in DKC patients (*P* = 3.07E-08), but overall also in AA patients (*P* = 0.0232). **C.** Delta telomere length was plotted against DNAm in *PRDM8* for DKC and AA samples. Cutoffs for 1 percentile in telomere length and 99 percentile in DNAm of *PRDM8* are indicated by areas shaded in blue.

Subsequently, we correlated DNAm in *PRDM8* with telomere length measurements. All DKC patients revealed telomere length below the 1% percentile in lymphocytes, as measured by flow-FISH, because such telomere attrition has been used as criterion for DKC diagnosis. In contrast, only 7.4% of AA patients (2 of 27) were below this threshold (Figure 3). Similar results were observed for granulocytes (Supplemental Figure 7). There was no correlation between telomere length and DNAm in *PRDM8.* Thus, the two biomarkers are independent and can therefore be considered as complementary for diagnosis of both bone marrow syndromes, particularly for those patients that do not reveal significant telomere attrition.

Aberrant hypermethylation in *PRDM8* is indicative for DKC and AA, but it is not disease specific. Notably, it was also observed in Down syndrome (DS) - another disease associated with symptoms of premature aging (Supplemental Figure 8) [23]. Analysis in The Cancer Genome Atlas (TCGA) demonstrated that the relevant genomic region was generally higher methylated in tumor tissue (Supplemental Figure 9). We reasoned that DNAm at *PRDM8* might be co-regulated with other genomic regions. Using 62 DNAm profiles of acute myeloid leukemia (AML) [24] we identified 151 CpGs showing clear correlation with beta-values at cg27242132 (Pearson correlation: *r* > 0.60 or *r* < - 0.60). Among these several CpGs corresponded to *PRDM16* indicating that the two functionally related genes might also be co-regulated on epigenetic level (Supplemental Figure 10) [25].

## CONCLUSIONS

Age-associated DNAm patterns are not generally accelerated in blood of DKC patients. However, we demonstrate that specific genomic regions reveal aberrant DNAm patterns in this disease. Aberrant hypermethylation was particularly observed at an internal promoter region of *PRDM8*. Notably, aberrant hypermethylation at the very same genomic region was also observed in patients with AA or Down syndrome - and all of these diseases present clinical features of premature aging. DNAm changes are also reflected on gene expression level and this warrants further functional studies in the future on whether or not PRDM8 directly impacts on cellular aging. On the other hand, we provide simple and cost-effective assays for DNAm measurements in *PRDM8* that are even applicable in unsorted peripheral blood. A variety of scoring systems based on TL in different cellular subsets have been proposed to improve diagnosis of AA and DKC [26], but it remains a challenge for patients without severe telomere attrition. DNAm in *PRDM8* should be further analyzed in such subsets of patients, in additional cohorts, and other diseases to ultimately demonstrate the clinical potential as a new biomarker.

## MATERIALS AND METHODS

### Samples of DKC and AA patients

Blood samples were obtained from the Registry for Telomeropathies and Aplastic Syndromes of RWTH Aachen University and participating hospitals (15 DKC patients and 32 AA patients). Furthermore, we used blood samples of 14 healthy individuals. The study was approved by the local ethic committee and all samples were taken after written consent (EK206/09). The DKC patients revealed TL below 1% percentile and diagnosis was complemented by clinical and genotypic characteristics (Supplemental Table 3). Clinical information about AA patients is provided in Supplemental Table 4.

### Next-generation sequencing of DKC patients

The genotype of four patients has already been published before [27-29]. For the other patients we utilized a multiplex-PCR approach (Truseq Amplicon, Illumina^®^) if enough DNA was available. To this end, we analyzed 158 amplicons covering 8 telomerase associated genes (*NOP10, NHP2, CTC1, DKC1, TERT, TERC, TIN2, RTEL1*). 250ng of genomic DNA of 9 DKC samples was prepared according to the TruSeq sample preparation guide. Sequencing data were analyzed using BaseSpace online analysis tool (Illumina, San Diego, CA, USA) and SeqPilot software (JSI medical systems, Ettenheim, Germany). To detect heterozygous or homozygous variants, conditions for scoring a mutant allele were: absolute coverage at SNV-Site ≥ 50 and relative allele burden of the variant ≥ 30% of all reads. Further analysis was performed using MutationTaster software (http://www.mutationtaster.org) and PolyPhen-2 software (http://genetics.bwh.harvard.edu/pph2/bgi.shtml) to predict pathogenicity of detected variants.

### Telomere length measurement by flow-FISH

Telomere length was analyzed as described previously [12, 30, 31]. Measurements were performed in triplicates with and without Alexa488-(C3TA2) PNA (Panagene, South Korea). Cow thymocytes were used as an internal control to calculate telomere length in kilo bases (kb). Identification of lymphocytes, granulocytes and cow thymocytes was done based on forward scatter properties and LDS 751 fluorescence. Peripheral blood of 104 healthy controls was used as for age-adaption as described previously [12, 30].

### DNA methylation profiles

Genomic DNA was isolated from four DKC patients with the QIAamp DNA Blood Midi Kit (Qiagen, Hilden, Germany; these samples are indicated in Supplemental Table 3). DNA quality was assessed with a NanoDrop ND-1000 spectrometer and by gel electrophoresis and then analyzed with the Illumina HumanMethylation450 BeadChip (Illumina). This platform can assay more than 480,000 CpG sites at single base resolution (covering 99% of RefSeq genes and 96% of CpG islands) [32]. Hybridization and initial data analysis using the BeadStudio Methylation module was performed at the DKFZ Gene Core Facility (Heidelberg, Germany). Raw data are available at Gene Expression Omnibus under the accession number GSE75310. Beta-values ranging from 0 (non-methylated) to 1 (100% methylation) are provided for each CpG site.

In addition, we utilized the following publically available datasets of normal peripheral blood: GSE32148 [33], GSE30870 [34], GSE36064 [35], GSE40005, GSE40279 [36], GSE41169 [37], GSE42861 [38], GSE50660 [39], GSE51180 [40], GSE51388, GSE56046 [41], GSE56105 [42], GSE56581 [41], GSE58651 [43], GSE61496 [44], GSE62992, and GSE49064 [45]. DNAm profiles of patients with Down syndrom were retrieved from GSE52588 [23]. AML profiles were used from a dataset by Qu et al. (GSE58477) [24]. Additional DNAm values were downloaded from the UCSC Cancer Genomics Browser (www.genome-cancer.ucsc.edu/) for various types of cancer as indicated. Unpaired limma *t*-test was calculated in R to select for significantly changed CpG sites (adjusted *P* value < 0.05).

### Pyrosequencing of *PRDM8*

Site-specific analysis of DNAm levels in *PRDM8* was analyzed for 14 controls, 13 DKC, and 20 AA samples. Primers were designed for two different regions (assay 1 and assay 2). 100 ng of DNA was bisulfite converted using the EZ DNA Methylation Kit (Zymo, Irvine, CA, USA) and amplified by PCR with annealing temperatures of 56°C and 65°C, respectively. After amplification 20 μl of the PCR product was immobilized to 5 μl of Streptavidin beads (GE Healthcare, Piscataway, NJ, USA) and annealed to 0.8 μl sequencing primer (20 μM) for 2 min at 80°C. For pyrosequencing the PyorMark Q96 ID system was utilized and analysis was performed with the PyroMark Q CpG software (Qiagen, Hilden, Germany). Primers: assay 1 forward: 5′-biotin-GGGGTTGTTT ATTGTTAGTA ATATTGTATA AAAGGAGGA-3′; assay 1 reverse: 5′-ACCCCGCTCT AAACCCAAAT TCTT-3′; assay 1 sequencing: 5′-GCCTACCCTA AAAATATACC-3′; assay 2 forward: 5′-AGTGAAACGG GGAAAGGTTT TTTTTAATTA TTTTGG-3′; assay 2 reverse: 5′-biotin-GTCCCCTCCC TTTAACTCTT TACTAAACCA A-3′; assay 2 sequencing 5′-TTTTGAGAGG CGTTGTTATT-3′.

### MassArray of *PRDM8*

Alternatively, we used MassARRAY for site-specific analysis of DNAm levels. This highly sensitive method is based on MALDI-TOF mass spectrometry and measurements were performed at Varionostic GmbH (www.varionostic.de; Ulm, Germany). Samples of 10 healthy controls, 11 DKC, and 27 AA patients were analyzed. Amplicons were designed with the Sequenom's EpiDESIGNER software. 150 ng of DNA was bisulfite converted with the EZ DNA Methylation Gold Kit (Zymo). Converted DNA was amplified by PCR using the HotStart Plus PCR Master Mix (Qiagen). Unincorporated dNTPs were neutralized using shrimp alkaline phosphatase (Agena Bioscience, San Diego, CA, USA). Subsequently, 10 μl of PCR product was *in vitro* transcribed and cleaved in a base-specific (U-specific) manner using RNase A (T-Cleavage MassCleave Kit; Agena Bioscience). The cleaved products were then analyzed by the MALDI-TOF mass spectrometer. Mass differences of 16 Da or n-fold were representative for methylation events. The experiment was performed with a MassARRAY Analyzer 4 System (Agena Bioscience). Primers: assay 1 forward: 5′-aggaagagag TTTTTGAGGG GTTGTTTATT GTTAGT-3′; assay 1 reverse: 5′-cagtaatacg actcactata gggagaaggc tTACCCTAAA AATATACCCC AAAACC-3′; assay 2 forward: 5′-aggaagagag GGGGAAAGGT TTTTTTTAAT TATTTT-3′; assay 2 reverse: 5′-cagtaatacg actcactata gggagaaggc tCCCTCCCTT TAACTCTTTA CTAAACC-3′ (Bases that correspond to the converted sequence of PRDM8 are indicated in capital letters).

### qRT-PCR of *PRDM8*

Expression of *PRDM8* was analyzed by real-time quantitative PCR (RT-qPCR) using the StepOneTM Instrument (Applied Biosystems, Applera Deutschland GmbH, Darmstadt, Germany). 250ng RNA was reverse-transcribed using the high capacity cDNA Reverse Transcription Kit (Applied Biosystems). cDNA was amplified using Power SYBR Green PCR Master Mix (Applied Biosystems). Gene expression was normalized to *GAPDH*. The following primers (Metabion, Martinsried, Germany) were used for amplification of *PRDM8*: forward: 5′-ACCAGCGTTT ACACCACCTG-3′; reverse: 5′-CCATTTGCTG CTGAGGTGTC-3′.

## SUPPLEMENTARY MATERIAL TABLES AND FIGURES




