# Supplementary material for: DNA methylation in PRDM8 is indicative for dyskeratosis congenita
Source: Oncotarget. 2016 Feb 17;7(10):10765–72. doi: 10.18632/oncotarget.7458 (PMC4905437; doi:10.18632/oncotarget.7458)
Supplement: Supplementary file 1 [file oncotarget-07-10765-s001.pdf]

DNA methylation in *PRDM8* is indicative for dyskeratosis congenita

Supplementary Material

Supplemental Table 1: Significantly changed CpG sites ($P < 0.05$).

(Provided as separate Excel file)

Supplemental Table 2: Highly differentially methylated CpG sites (cutoff 0.3).

CpG site	Gene symbol	Gene name	Fold change	Adjusted *P* value
19 CpG sites hypermethylated in DKC				
cg10306192	*MMP27*	matrix metallopeptidase 27	-0.50	7.52E-06
cg14179288	*TLE4*	transducin-like enhancer of split 4	-0.49	4.68E-05
cg27242132	***PRDM8***	PR domain containing 8	-0.47	2.56E-02
cg02898977	*TM9SF1*	transmembrane 9 superfamily member 1	-0.41	6.28E-06
cg11155924	*SHANK2*	SH3 and multiple ankyrin repeat domains 2	-0.41	4.18E-02
cg10493186	***PRDM16***	PR domain containing 16	-0.40	3.22E-02
cg07056794			-0.37	3.64E-04
cg02458885	***PRDM8***	PR domain containing 8	-0.37	3.60E-02
cg11808699	*IL16*	interleukin 16	-0.37	3.51E-02
cg26299084	***PRDM8***	PR domain containing 8	-0.36	4.53E-02
cg06307913	***PRDM8***	PR domain containing 8	-0.36	3.81E-02
cg03466780	*EXD3*	exonuclease 3'-5' domain containing 3	-0.35	1.50E-05
cg10296238	*SPATC1L*	spermatogenesis and centriole associated 1-like	-0.35	2.57E-02
cg08206623	*CDKN1C*	cyclin-dependent kinase inhibitor 1C	-0.34	7.95E-05
cg03131366			-0.33	4.90E-02
cg18073471	***PRDM8***	PR domain containing 8	-0.32	2.94E-02
cg25152348	*NCAPH2; LMF2*	non-SMC condensin II complex, H2; lipase maturation factor 2	-0.32	2.61E-04
cg09844907	*MPV17L*	MPV17 mitochondrial membrane protein-like	-0.32	3.64E-04
cg02610360	*TMEM136*	transmembrane protein 136	-0.31	7.71E-03
7 CpG sites hypomethylated in DKC				
cg14733031	*B3GNT3*	UDP-GlcNAc:betaGal beta-1,3-N-acetylglucosaminyltransferase 3	0.39	2.96E-03
cg01775802	*RGS6*	regulator of G-protein signaling 6	0.37	2.28E-03
cg04205664	*CLEC2L*	C-type lectin domain family 2, member L	0.37	8.00E-04
cg15897635			0.35	9.51E-04
cg00695177			0.33	1.50E-05
cg24807850	***PRDM16***	PR domain containing 16	0.32	3.04E-02
cg21565415	*MUPCDH*	cadherin-related family member 5	0.31	4.58E-04

Supplemental Table 3: Clinical data of the patients with dyskeratosis congenita

ID	Sample ID on array	Age in years	Sex	Leucoplakia	Hypopigmentation	Nail dystrophy	Hair greying	Developmental delay	Cerebral hypoplasia	BM failure	Liver cirrhosis	Lung fibrosis	Family history	TL <1%	Genotype
1		6	M	N	N	N	N	N	N	Y	N	N	Y	Y	TERT, T1129P*
2		20	M	Y	N	Y	Y	N	N	Y	N	N	Y	Y	TERC, C116T**; ***
3	DKC1	16	F	N	N	Y	N	N	N	Y	N	N	Y	Y	TERC, C116T**
4		31	M	Y	Y	Y	Y	N	Y	N	Y	Y	Y	Y	DKC1, R449Q
5		19	M	N	Y	N	N	N	N	Y	N	Y	Y	Y	TERC,c.2372T>C
6		42	M	N	Y	N	N	N	N	Y	N	N	Y	Y	TERC,c.2372T>C
7	DKC3	3	F	Y	N	Y	N	Y	Y	Y	Y	Y	N	Y	N.D.
8		34	M	N	Y	N	Y	N	N	Y	N	N	N	Y	No mutation identified
9		34	F	Y	N	N	N	N	N	Y	N	N	N.K	Y	TERT, 1258A>C
10		5	M	Y	N	N	N	N	N	Y	N	N	N	Y	RTEL1,c.1346T>C
11		22	F	Y	Y	Y	N	N	N	Y	Y	N	N	Y	TERC r.128 A>G
12		28	F	N	N	N	Y	N	N	Y	N	Y	N.K	Y	N.D.
13		27	M	Y	Y	N	Y	N	N	Y	Y	N	Y	Y	No mutation identified
14	DKC2	2	F	N	N	N	N	Y	Y	Y	N	N	N	Y	TIN2, R282H
15	DKC4	10	F	N	N	N	N	N	N	Y	N	N	Y	Y	TERT, T1129P*

The four samples that were used for DNAm profiling are indicated (DKC1-4). For comparison we utilized four gender-matched controls of similar age that were obtained from a dataset by Harris et al. 2012 (GSE32148: sample IDs: GSM796678; GSM796674; GSM796667; GSM796671). Some of the DKC samples have also been described in original publications as indicated (*[1]; **[2]; ***[3]).

Supplemental Table 4: Clinical data of aplastic anemia patients

Clinical parameters	Aplastic anemia patients (n=32)
Age in years ± SD	40.9 ± 19,3
Sex	45% (n=14) female / 56% (n=18) male
Classification	
○ Moderate aplastic anemia	19% (n=6)
○ Severe aplastic anemia	53% (n=17)
○ Very severe aplastic anemia	28% (n=9)
Treatment	
○ CSA mono	9% (n=3)
○ CSA/ATG	50% (n=16)
○ Frontline allogeneic transplantation	3% (n=1)
○ Others/not available	38% (n=12)

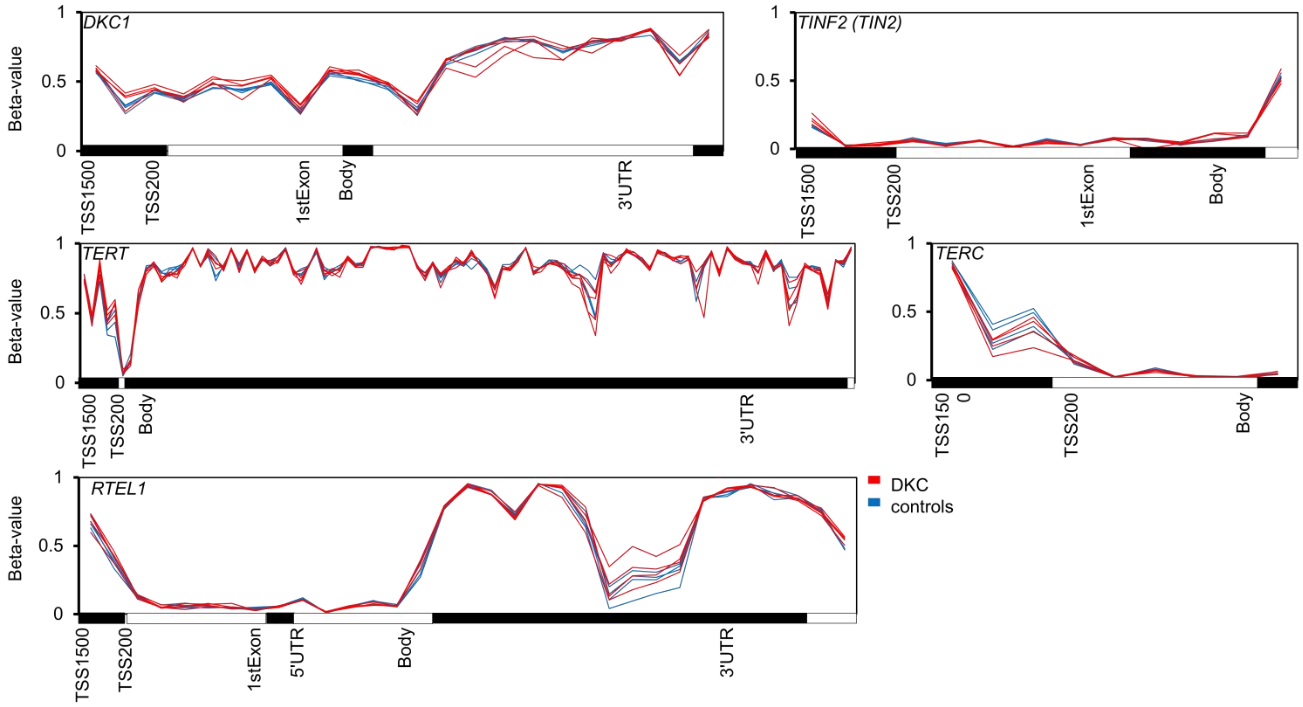

Supplemental Figure 1: DNAm profiles of DKC disease related genes.

Mutations in the following genes have been causally linked to DKC: dyskeratosis congenita 1, dyskerin (*DKC1)*, TERF1 (TRF1)-interacting nuclear factor 2 (*TINF2)*, telomerase reverse transcriptase (*TERT*), telomerase RNA component (*TERC)*, and regulator of telomere elongation helicase 1 (*RTEL1)*. The plots depict the beta-values of all corresponding CpGs that are linked to these genes on the HumanMethylation450 BeadChip. The DNAm patterns did not reveal significant differences in these genes as compared to control samples (four age and gender matched samples are exemplarily depicted: GSM796678; GSM796674; GSM796667; GSM796671).

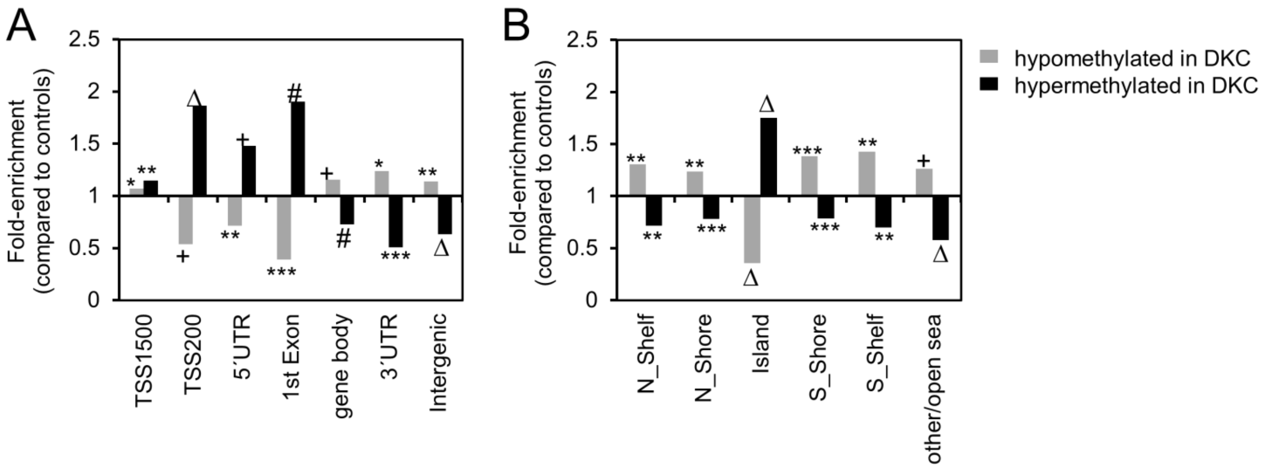

Supplemental Figure 2: Enrichment of DNAm changes in genomic regions.

The annotations of CpG sites to either regions of corresponding genes (**A;** TSS1500: 1500 bp up-stream of transcription start site; TSS200: 200 bp upstream of TSS; UTR: untranslated region) or to CpG islands **(B)** [4] were used to estimate enrichment of DNAm changes at specific regions (hypergeometric distribution; $^{*}P < 0.05$; $^{**}P < 0.01$; $^{***}P < 0.001$; $^{+}P < 10^{-5}$; $^{\#}P < 10^{-10}$; $^{\Delta}P < 10^{-15}$). Hypermethylated sites are highly significantly enriched in promoter regions and CpG islands.

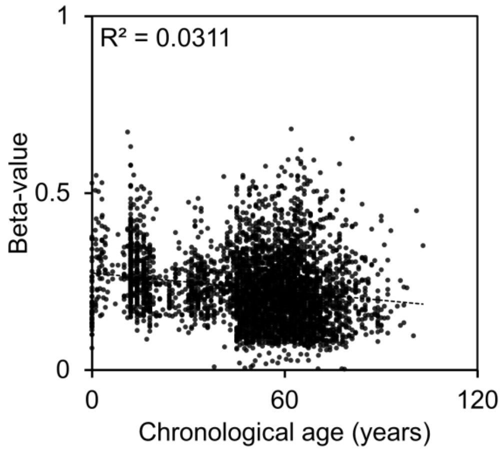

Supplemental Figure 3: DNA-methylation levels in *PRDM8* are independent of donor age.

Beta-values were retrieved form DNAm profiles of 4,131 healthy blood samples of 16 different studies (GSE32148, GSE30870, GSE36064, GSE40005, GSE40279, GSE41169, GSE42861, GSE50660, GSE51180, GSE51388, GSE56046, GSE56105, GSE56581, GSE58651, GSE61496, and GSE62992). The results for the CpG site cg27242132 are exemplarily depicted (results are similar for neighboring CpGs) – these CpGs did not reveal clear association with chronological age.

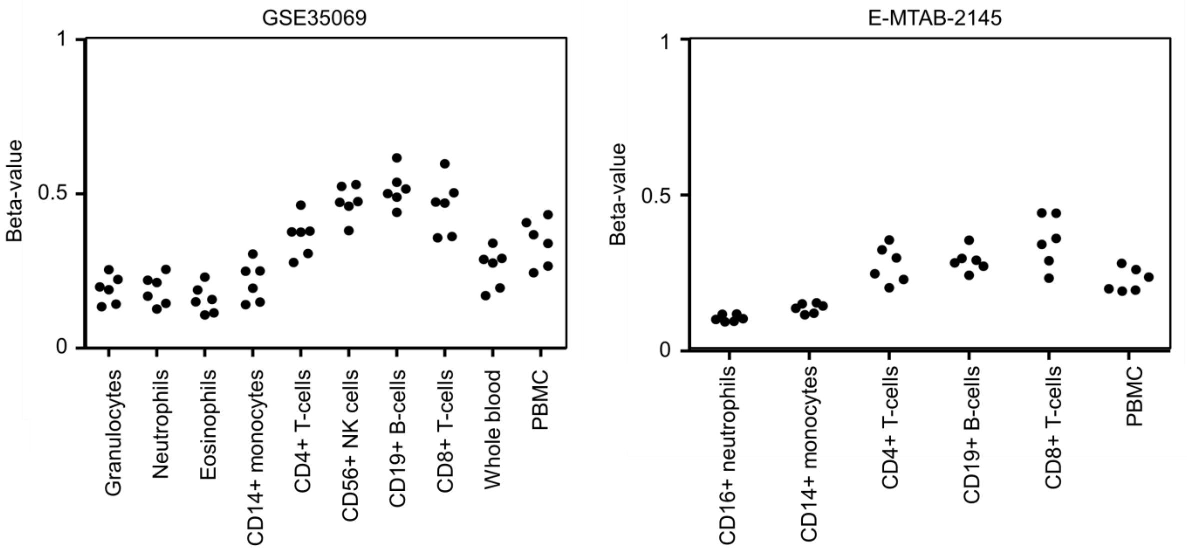

Supplemental Figure 4: Lymphocytes reveal slightly higher DNAm in *PRDM8*.

To estimate the impact of subcellular composition on the DNAm levels in *PRDM8* we utilized DNAm profiles of two studies that analyzed sorted cell populations (GSE35069, E-MTAB-2145) [5,6]. Beta-values for the CpG site cg27242132 are depicted. There was a moderate increase in DNAm levels in lymphocytes indicating that cell counts in blood samples have some impact on DNAm measurements in blood samples at this region.

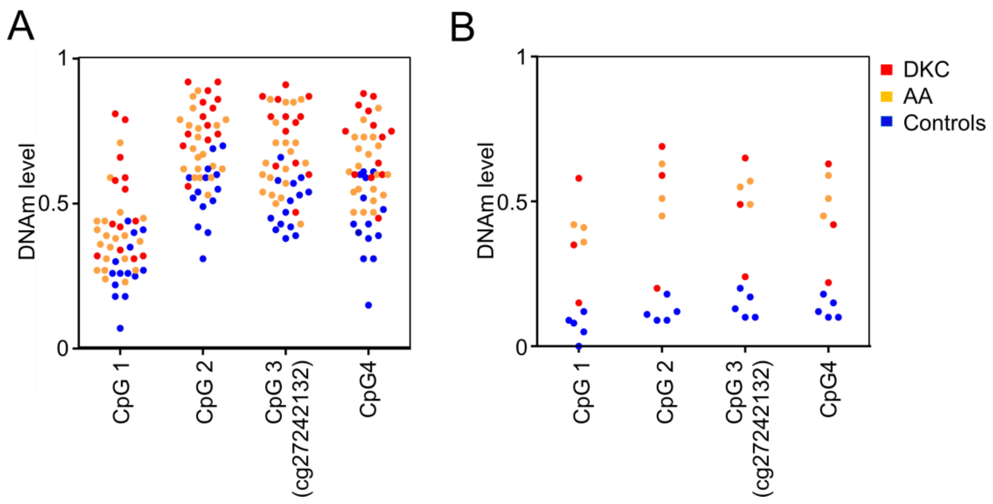

Supplemental Figure 5: Pyrosequencing assays in *PRDM8* are temperature sensitive.

This graphic depicts pyrosequencing measurements of DNAm levels at cg27242132 in DKC, AA, and healthy controls. The PCR on bisulfite converted DNA was either performed with an annealing temperature of **(A)** 65 °C or **(B)** 56 °C. Generally, the results are in line with the observations of the HumanMethylation450 BeadChips. However, the absolute DNAm levels were affected by temperature settings (similar results were also observed for assay 2). Therefore, we reasoned this approach might be less suitable for quantitative analysis as diagnostic biomarker and alternatively focused on MassARRAY.

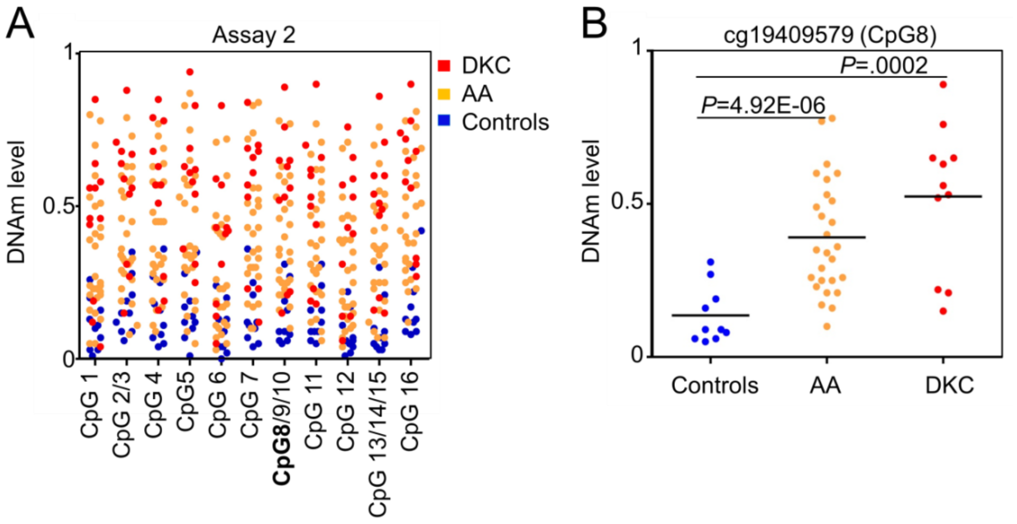

Supplemental Figure 6: MassARRAY analysis of DNAm at cg19409579 (assay 2).

(A) MassARRAY analysis the region with CpG site cg19409579 (corresponds to CpG8 in assay 2) demonstrated that neighboring CpGs reflect similar DNAm levels. Furthermore, the DNAm levels corresponded very well to beta-values of the HumanMethylation450 BeadChip. **(B)** Overall, AA (n = 27) and DKC (n = 11) revealed significantly higher DNAm levels than controls (n = 10; two-sided Student's Test).

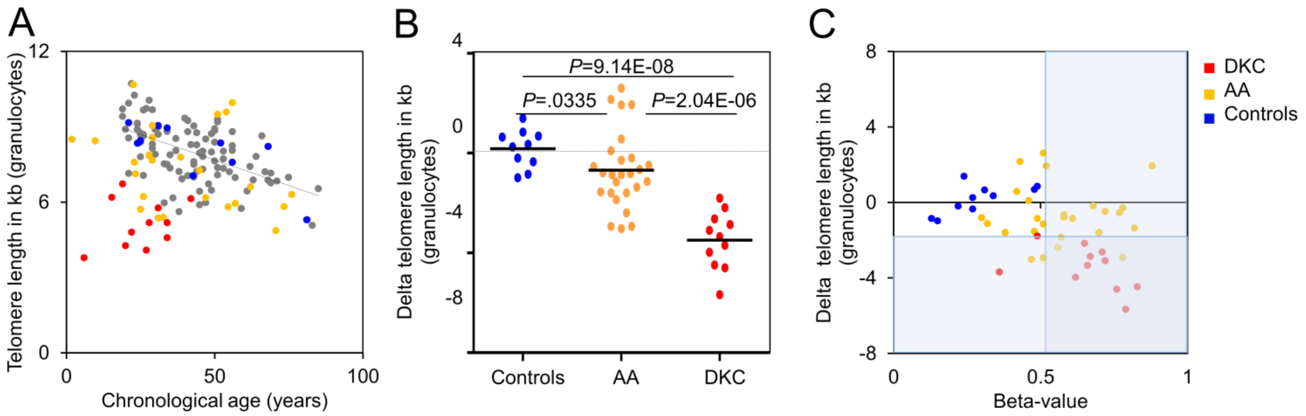

Supplemental Figure 7: Telomere length analysis in granulocytes.

(A) Telomere length of granulocytes was analyzed by Flow-Fish for 104 healthy control samples (grey and blue),[7] AA (yellow) and DKC (red). **(B)** Telomere length was then analyzed in relation to the mean age-adjusted telomere length of 104 control samples (delta telomere-length): AA and particularly DKC revealed significant telomere attrition. Statistical significance was estimated by two-sided Student's Test. **(C)** DKC diagnosis and discrimination of AA samples was supported by setting a cutoff of <1 percentile of telomere length and > 99 percentile DNAm (DNAm = 0.52; shaded in blue).

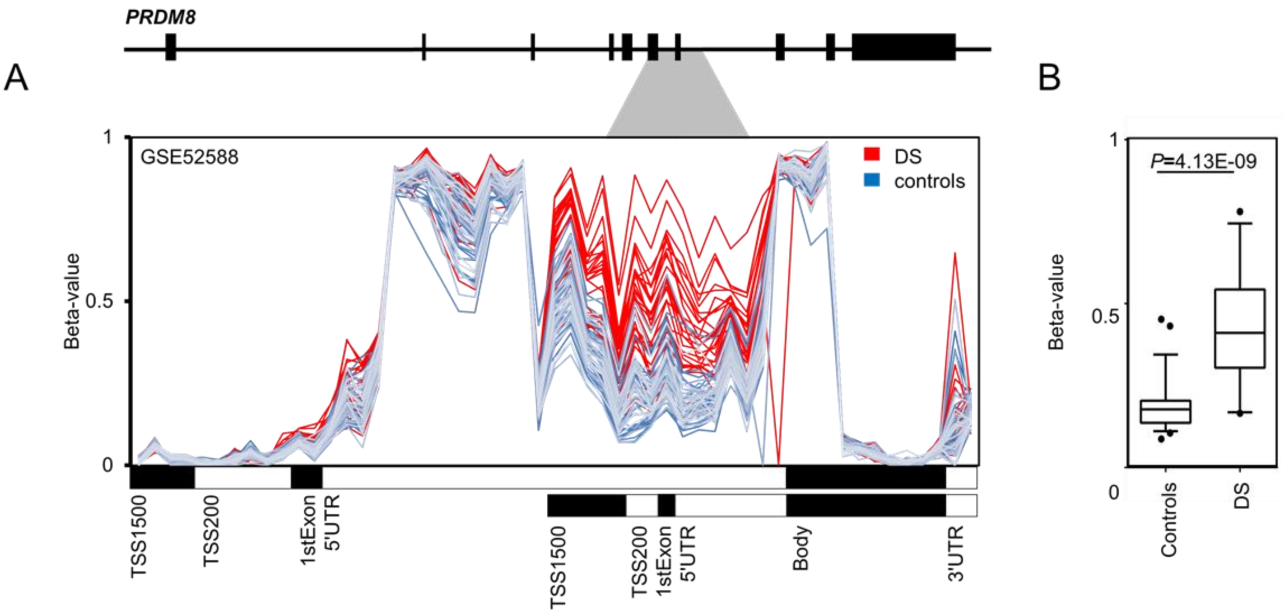

Supplemental Figure 8: Blood of Down syndrome patients reveals hypermethylation in *PRDM8*.

(A) DNA-methylation profiles of Down syndrome patients (DS; n = 29) revealed significantly higher DNAm at the internal promoter region of *PRDM8* as compared to controls (n = 58) [8]. Beta-values are depicted for all CpGs associated with *PRDM8* (in analogy to Figure 2B). (B) Direct comparison of beta-values at the CpG site cg27242132 (two-sided Student's Test: *P* = 4.13E-09).

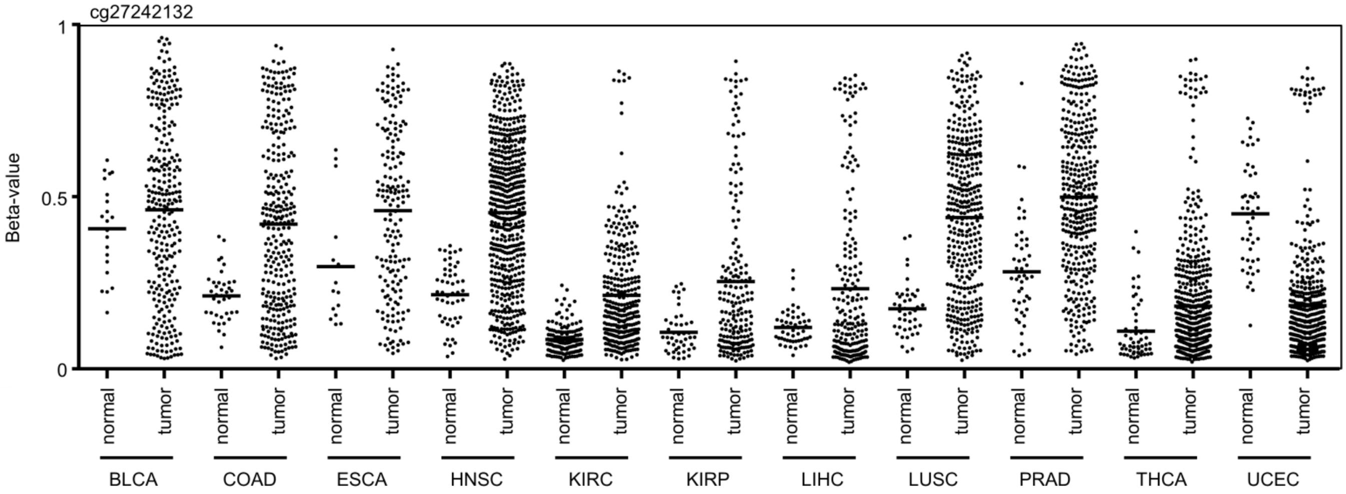

Supplemental Figure 9: DNAm in *PRDM8* (cg27242132) in different types of cancer.

DNAm level at the CpG site cg27242132 was analyzed using the UCSC Cancer Genomics Browser (https://genome-cancer.ucsc.edu/). Except of UCEC, all tumor types depicted an increased DNAm compared to normal tissue controls. BLCA = bladder urothelial carcinoma, COAD = colon adenocarcinoma, ESCA = esophageal carcinoma, HNSC = head and neck squamous cell carcinoma, KIRC = kidney renal clear cell carcinoma, KIRP = kidney renal papillary cell carcinoma, LIHC = liver hepatocellular carcinoma, LUSC = lung squamous cell carcinoma, PRAD = prostate adenocarcinoma, THCA = thyroid carcinoma, UCEC = uterine corpus endometrial carcinoma.

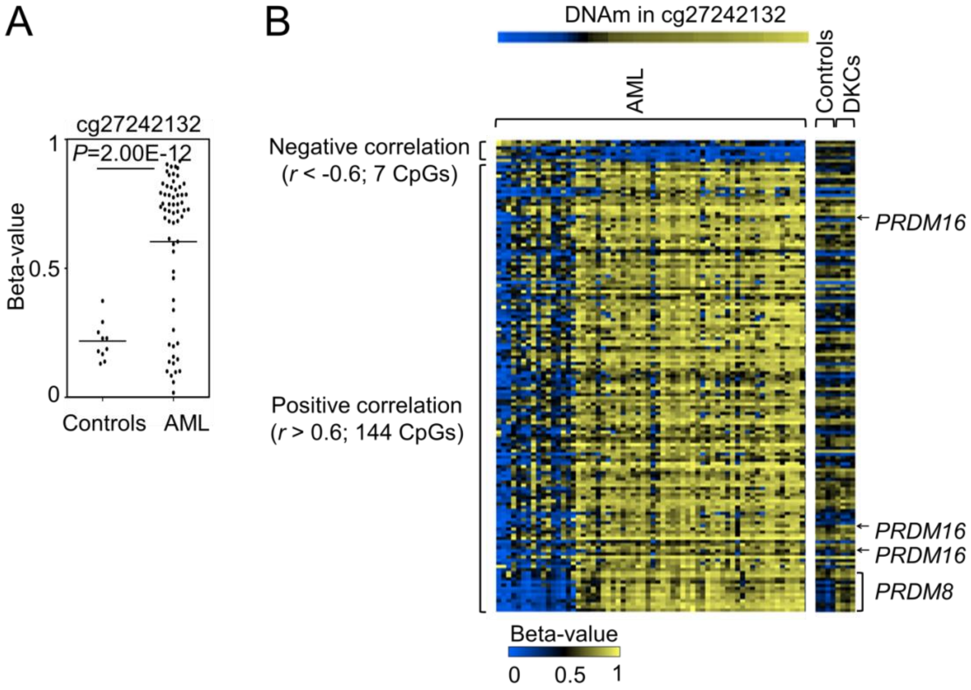

Supplemental Figure 10: Correlation of DNAm at cg27242132 with other CpG sites in AML.

To identify CpGs that might be co-regulated with DNAm at *PRDM8* we utilized a dataset of 62 DNAm profiles of AML (GSE58477 [9] due to low variation such analysis would not be possible in normal blood). **(A)** Overall, AML depicted significantly higher DNAm levels in cg27242132 than healthy controls (P = 2.00E-12). **(B)** Then, we focused on CpGs that correlate linearly in their DNAm level with cg27242132 (Pearson correlation coefficient of $r > 0.60$ or $r < -0.60$): seven CpGs were anti-correlated, whereas 144 CpGs revealed a positive correlation. As expected, several CpGs in *PRDM8* correlated with DNAm at cg27242132. Interestingly, the same was observed for several CpGs in *PRDM16* indicating that epigenetic regulation of the two genes might be related. Notably, two CpGs in *PRDM16* revealed also aberrant DNAm in DKC (Supplemental table 2).

References of Supplementary Data

1. *Stockklausner C., Raffel S., Klermund J., Bandapalli O. R., Beier F., Brummendorf T. H., Burger F., Sauer S. W., Hoffmann G. F., Lorenz H., Tagliaferri L., Nowak D., Hofmann W. K., et al. A novel autosomal recessive TERT T1129P mutation in a dyskeratosis congenita family leads to cellular senescence and loss of CD34+ hematopoietic stem cells not reversible by mTOR-inhibition. Aging (Albany NY) 2015; 7: 911-927.*
2. *Trautmann K., Jakob C., von G. U., Schleyer E., Brummendorf T. H., Siegert G., Ehninger G., Platzbecker U. Eltrombopag fails to improve severe thrombocytopenia in late-stage dyskeratosis congenita and diamond-blackfan-anaemia. Thromb Haemost 2012; 108: 397-398.*
3. *Stepensky P., Rensing-Ehl A., Gather R., Revel-Vilk S., Fischer U., Nabhani S., Beier F., Brummendorf T. H., Fuchs S., Zenke S., Firat E., Pessach V. M., Borkhardt A., et al. Early-onset Evans syndrome, immunodeficiency, and premature immunosenescence associated with tripeptidyl-peptidase II deficiency. Blood 2015; 125: 753-761.*
4. *Bibikova M., Barnes B., Tsan C., Ho V., Klotzle B., Le J. M., Delano D., Zhang L., Schroth G. P., Gunderson K. L., Fan J. B., Shen R. High density DNA methylation array with single CpG site resolution. Genomics 2011; 98: 288-295.*
5. *Reinius L. E., Acevedo N., Joerink M., Pershagen G., Dahlen S. E., Greco D., Soderhall C., Scheynius A., Kere J. Differential DNA methylation in purified human blood cells: implications for cell lineage and studies on disease susceptibility. PLoS ONE 2012; 7: e41361.*
6. *Zilbauer M., Rayner T. F., Clark C., Coffey A. J., Joyce C. J., Palta P., Palotie A., Lyons P. A., Smith K. G. Genome-wide methylation analyses of primary human leukocyte subsets identifies functionally important cell-type-specific hypomethylated regions. Blood 2013; 122: e52-e60.*
7. *Weidner C. I., Lin Q., Koch C. M., Eisele L., Beier F., Ziegler P., Bauerschlag D. O., Jockel K. H., Erbel R., Muhleisen T. W., Zenke M., Brummendorf T. H., Wagner W. Aging of blood can be tracked by DNA methylation changes at just three CpG sites. Genome Biol 2014; 15: R24.*
8. *Bacalini M. G., Gentilini D., Boattini A., Giampieri E., Pirazzini C., Giuliani C., Fontanesi E., Scurti M., Remondini D., Capri M., Cocchi G., Ghezzo A., Del R. A., et al. Identification of a DNA methylation signature in blood cells from persons with Down Syndrome. Aging (Albany NY) 2015; 7: 82-96.*
9. *Qu Y., Lennartsson A., Gaidzik V. I., Deneberg S., Karimi M., Bengtzen S., Hoglund M., Bullinger L., Dohner K., Lehmann S. Differential methylation in CN-AML preferentially targets non-CGI regions and is dictated by DNMT3A mutational status and associated with predominant hypomethylation of HOX genes. Epigenetics 2014; 9: 1108-1119.*
